# M13KO7 bacteriophage enables Potato Virus Y detection

**DOI:** 10.1128/spectrum.01446-23

**Published:** 2023-10-09

**Authors:** Haneul Seo, Sang-Ho Cho, Thuy T. B. Vo, Ahlim Lee, Sungrae Cho, Sol Kang, Eui-Joon Kil, Hee-Seong Byun, Mi-Gi Lee, Myung-Hee Kwon, Woo-Jae Chung, Young-Gyu Lee, Sukchan Lee

**Affiliations:** 1 Department of Integrative Biotechnology, Sungkyunkwan University, Suwon, Republic of Korea; 2 Department of Plant Medicals, Andong National University, Andong, Republic of Korea; 3 Crop Protection Division, National Academy of Agricultural Science, Rural Development Administration, Wanju, Republic of Korea; 4 Biocenter, Gyeonggido Business & Science Accelerator, Suwon, Republic of Korea; 5 Department of Microbiology, Ajou University School of Medicine, Suwon, Republic of Korea; 6 Highland Agriculture Research Institute, National Institute of Crop Science, Rural Development Administration, Pyeongchang, Republic of Korea; 7 Department of Biopharmaceutical Convergence, School of Pharmacy, Sungkyunkwan University, Seoul, Republic of Korea; Fujian Agriculture and Forestry University, Fuzhou City, Fujian, China

**Keywords:** *Potato virus Y*, M13 bacteriophage, ELISA, virus interaction, virus detection

## Abstract

*Potato virus Y* (PVY) is one of the foremost plant viruses affecting potato plants and production. Many diagnostic and detection techniques are available to detect and prevent the spread of this virus. Serological analyses, such as enzyme-linked immunosorbent assay (ELISA), are widely used in laboratories as well as in the field. In addition, recently, serological methods using whole or recombinant antibodies have been developed. However, these antibodies are unstable under certain chemical conditions and can be easily degraded by exposure to enzymes. M13KO7, a derivative of bacteriophage M13, with a filamentous structure similar to PVY, was used for antibody screening. We found that PVY and “bald” M13KO7 had binding activity similar to that of antigen-antibody in ELISA analyses. The binding interactions between PVY and M13KO7 were highly specific. A new method for detecting PVY using ELISA analysis with M13KO7 was developed in this study. These results provide new insights into two virus-binding interactions and novel systems for the detection of plant viruses.

**IMPORTANCE:**

In this study, we confirmed the binding of M13KO7 to *Potato virus Y* (PVY) using enzyme-linked immunosorbent assay. M13KO7 is a “bald” bacteriophage in which no recombinant antibody is displayed. M13KO7 is easy to propagate by using *Escherichia coli*, making this method more reasonable in economic perspective. Based on this study, we suggest that M13KO7 detection system has applicability as a novel biological tool for the detection of PVY

## INTRODUCTION

Cultivated potatoes are an important global food crop and arguably one of the most intensively managed crops, requiring appropriate irrigation, fertilization, and frequent pesticide application to obtain competitive yields (1). Since potatoes are cultivated via vegetative propagation, their production differs remarkably from that of crops grown from true seeds, creating unique opportunities for the propagation and spread of diseases, such as those caused by viruses (2). The incidence and intensity of viral infections occurring alongside vegetative propagation are known to increase with successive propagations, resulting in an uneconomical yield after 3–4 y.

*Potato virus Y* (PVY) is a member of the Potyviridae family of plant viruses (3, 4). It has long filamentous particles that are 750 nm long and 11 nm wide. Its genome consists of a single-stranded positive-sense RNA that is translated to form a single polyprotein with eight cleaved proteins (5). PVY is the most serious potato virus known to decrease crop yields (6, 7). Plant viruses such as PVY are difficult to treat; therefore, their early diagnosis and prevention of spread are crucial (8). Among the potential ways to prevent the spread of plant viruses, serological techniques are frequently favored because of their specificity, speed, simplicity, and scope of standardization (9, 10). However, conventional serological techniques cannot be used for many important species because the target protein may not be expressed, making it difficult to obtain target-specific antigens (11, 12). In addition, antibodies are easily degraded by enzymes, pH, and heat (13).

The M13 bacteriophage, belonging to *Inoviridae*, is an enterobacteria bacteriophage that infects *Escherichia coli*. This bacteriophage has a different structure from others and a different method of release from infected cells. M13 bacteriophages consist of circular single-stranded DNA molecules encased in a thin flexible tube to form a filamentous structure with a length of approximately 900 nm and a diameter of 7 nm (14, 15). M13 structure is highly resistant to heat and is stable against chemical or mechanical stresses, even non-polar organic solvents (16 – 18). One M13 derivative, the M13KO7 bacteriophage, contains the kanamycin resistance gene *Tn903*, inserted at the M13 origin of replication (19, 20). Recombinant M13 bacteriophages are frequently used in bacteriophage displays for antibody screening (19, 21 – 24). M13KO7 can replicate in the absence of phagemid DNA and has a heat-resistant robust structure (22).

In this study, we confirmed the binding of M13KO7 to PVY using ELISA. M13KO7 is a “bald” bacteriophage in which no recombinant antibody is displayed. We identified the detection specificity using several potyviruses obtained from the Plant Virus GenBank (PVGB) (Republic of Korea). This study further suggested that the molecular structures of M13KO7 and PVY are involved in this binding interaction.

## RESULTS

### M13 bacteriophage ELISA detection for PVY

**Fig 1:**
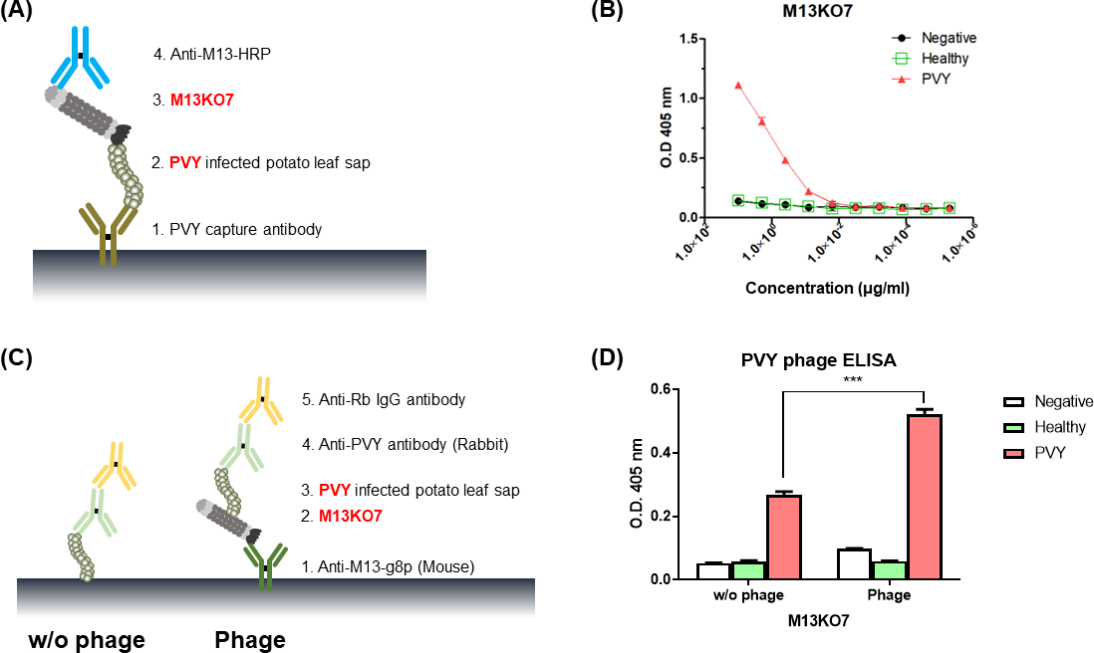
Schematic views and results of bacteriophage ELISA. (**A**) Schematic of an indirect sandwich ELISA for detection of Potato virus Y (PVY) by M13KO7. (**B**) Results of PVY detection bacteriophage ELISA measured at OD_405_ using a spectrophotometer. M13KO7 bacteriophages were serial diluted from 10 to 5.12 µg/mL. (**C**) Schematic of reverse ELISA of (**A**). Coating M13 bacteriophages directly to the plates and binding with target antigens resulted in a lower signal. As such, anti-M13-g8p antibody was used for capturing bacteriophages. (**D**)The result of reverse ELISA was measured at OD_405_ using a spectrophotometer. PVY capture using M13KO7 was higher than that obtained by directly coating PVY on the plate. Two-tailed *t*-test was used to analyze the results of only PVY and bacteriophage-captured PVY (***P = 0.0002; GraphPad Prism, GraphPad).

M13KO7 bacteriophages were collected via bacteriophage propagation using XL1-Blue. The bacteriophage titer was approximately 7.0 × 10^11^ CFU/mL. Two different ELISA methods (25) were performed (Fig. 1A and C) to confirm the binding affinity of M13KO7 and PVY. The serially diluted M13KO7 showed binding affinity with PVY samples above 0.08 µg/mL (Fig. 1B) and showed no activity with negative and healthy samples. Secondary ELISA tests were performed as inverse assays for the first ELISA (Fig. 1C). Coating M13 bacteriophages directly to the plate and binding with target antigens has been shown to produce a lower signal (25). The bacteriophages were coated on the ELISA plates using the capture antibody, and PVY viruses were detected (Fig. 1D). In general, PVY can be directly coated onto a plate without using capture antibodies. Here, we confirmed that using M13KO7 increased the binding affinity of PVY. The bacteriophages or PVY were coated on the ELISA plate with the capture antibody, and the binding affinities of the two viruses were confirmed.

### Effect of thermal denaturation on M13KO7 ELISA for PVY

We investigated whether the denatured protein structure of M13KO7 by heat could affect PVY detection, which was investigated using M13KO7 denatured with heat for 30 min or normal bacteriophages. PVY binding affinity of the protein structure of M13KO7 was lost due to heat denaturation (Fig. S1A). As an extension of the MALDI results from the pH experiments, we also analyzed the MALDI spectrum of thermally denatured M13KO7 (Fig. S1B). The thermally denatured M13KO7 showed the same spectral pattern as the normal M13KO7, with a 5,239 *m*/*z* peak of the major coat protein PVIII. Based on previous research about differential scanning calorimetry profile of M13 phage, heat capacity of M13(Cp) consists of maximum at a 93°C and a shoulder extending from 97°C to almost 110°C, causing irreversible denaturation (26).

### Effect of pH on M13KO7 ELISA for PVY

**Fig 2:**
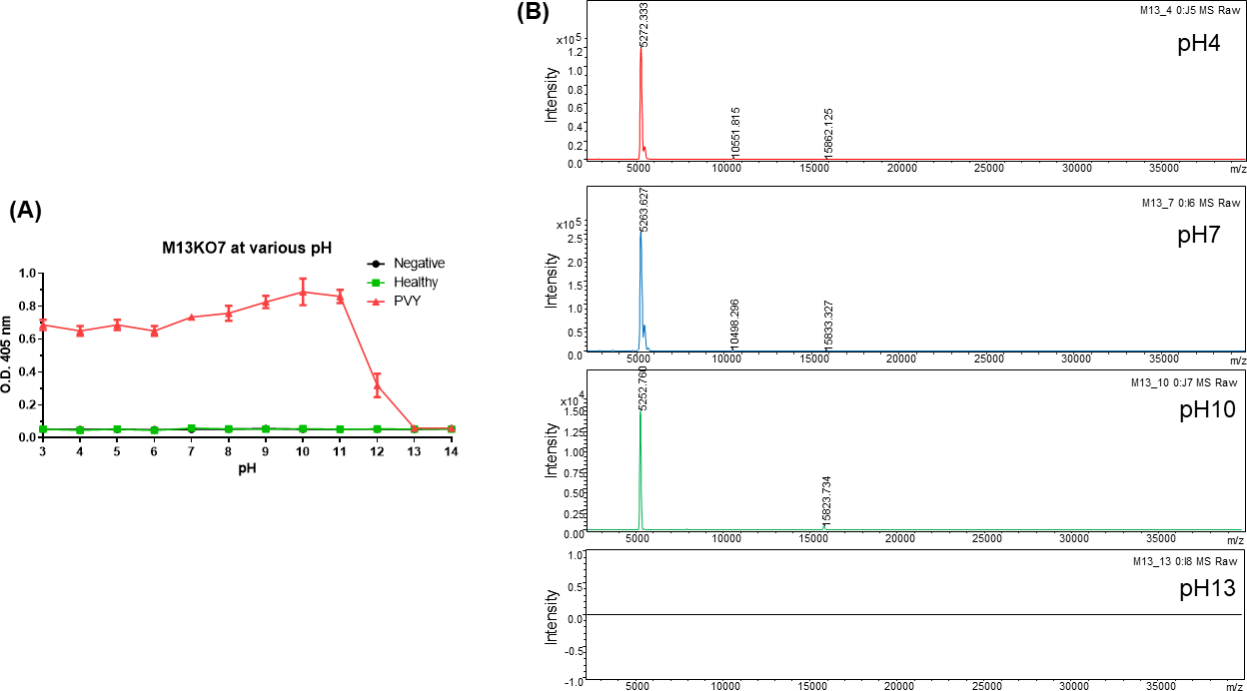
Effect of pH on M13KO7 ELISA for Potato virus (PVY). (**A**) Results of ELISA analysis of M13KO7 treated with buffers at various pH (pH 3‒14). (**B**) Analysis of MALDI-TOF spectral pattern of M13KO7 at different pH. A significant relationship is observed between the apparent peak in the spectrum and PVY detection in the specific pH range.

The effects of pH on 13KO7 detection ability were determined. After pretreatment of M13KO7 with a solution of pH 3‒14, the bacteriophages were diluted with a blocking buffer and the detection ability against PVY was confirmed (Fig. 2A). From pH 3 to 7, no significant change was observed in the binding ability of M13KO7 to PVY. The ability to bind specifically to PVY gradually increased from pH 8 to 11, after which it tended to decrease rapidly at pH 12 or higher. These results indicate that pretreating M13KO7 with specific base pH levels amplified the detection efficiency of PVY.

Matrix-assisted laser desorption/ionization-time-of-flight mass spectrometry (MALDI-TOF MS) analysis was performed to determine whether the changes with respect to pH were related to the structure of M13KO7 (Fig. 2B). The M13KO7 MALDI spectra at both pH 7 and pH 10, which were possible for PVY detection, showed similar patterns, including peaks for molecular weights of the coat proteins. The peak pattern at pH 12 changed, which was related to a lower PVY detection ability. At a high pH (pH 13 and 14), hydrolysis by sodium hydroxide occurred, and no peak was observed in the MALDI spectra. This result was consistent with the no-PVY detection in the ELISA. Taken together, the spectral results of MALDI indicated that the pH changes affected the proteins of M13KO7 influencing the binding affinity of PVY. Based on previous research, pH could affect the assembly and final ordering densities of M13 (27).

### Yeast two-hybrid results between PVY CP and M13KO7 CPs

**Fig 3:**
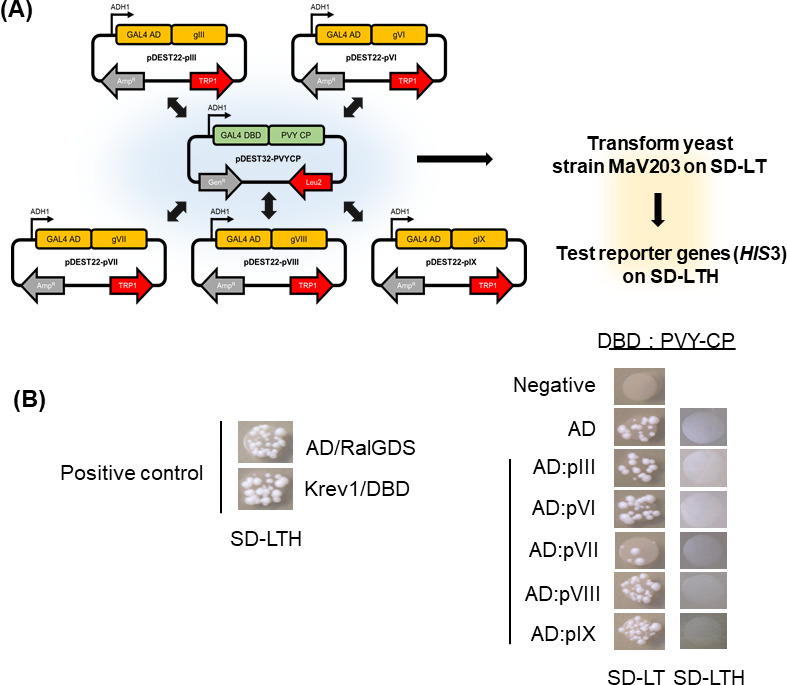
Yeast two-hybrid results between PVY CP and M13KO7 CPs. (**A**) The Y2H system is based on the principle that the transcription factor is composed of two domains, a DBD and an AD. Both hybrids are inserted into separate plasmids and have independent selection markers. Five coat proteins of M13KO7 were cloned into the pDEST22 plasmid (prey vector), and PVY CP into the pDEST32 plasmid (bait vector). Prey and bait were co-transformed into MaV203 in pairs and selected on SD-LT plates. MaV203 strain contains reporter genes (*HIS*3) for evaluating the hybridization of the proteins. (**B**) The validity of the Y2H system was confirmed by the formation of positive control colonies on SD-LTH plates (*HIS*3 reporter confirmation medium). RalGDS fused to the GAL4 DNA AD and Krev-1 (a member of GTP binding proteins) fused to the GAL4 DBD. Bait (PVY CP) and prey plasmids (M13KO7 CPs) were co-transformed into MaV203 yeast strain. All transformants except the negative control (non-transformant) formed colony on SD-LT plate. On the other hand, colonies did not appear in the LD-LTH.

Y2H analysis was performed to confirm that the binding affinity between PVY and M13KO7 is related to single coat protein interaction (Fig. 3A). Five coat proteins of M13KO7 (pIII, pVI, pVII, pVIII, and pIX) were cloned into the prey plasmid, and PVY CP was cloned into the bait plasmid. Yeast colonies were formed on SD-LT medium and transferred onto SD-LTH medium to confirm *HIS3* reporter gene expression (Fig. 3B). The positive controls, DNA activation domain with RalGDS (AD/RalGDS), and Krev-1 with a DNA-binding domain (Krev1/DBD) formed colonies on SD-LTH medium. In contrast, PVY CP and five coat proteins of M13KO7 CPs did not hybridize. These results indicated that any single coat protein of M13KO7 cannot bind with PVY coat protein individually. Based on this Y2H data, the interactions between PVY and M13KO7 were seemed to be caused by the whole structure-based bindings between PVY and M13KO7.

### Specific binding test of M13KO7 to PVY

**Fig 4:**
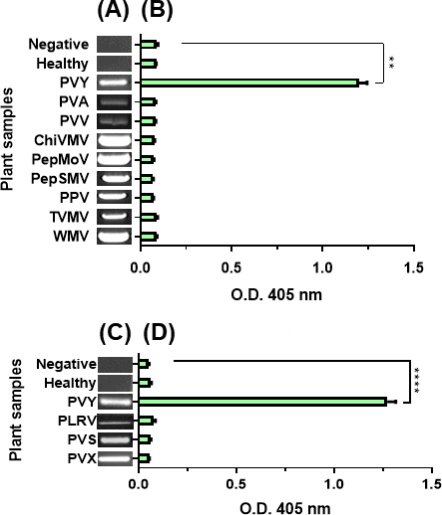
Plant virus detection. (**A**) Potyvirus RT-PCR result of potato sample. (**B**) ELISA of binding of M13KO7 to several potyviruses measured at OD_405_ using a spectrophotometer. The highest bacteriophage binding was only PVY. (**C**) Potato viruses RT-PCR result of potato sample. (**D**) ELISA result of M13KO7 binding to several potato viruses. The bar graph indicates the mean ± standard deviation (*n* = 3). The statistical comparison was performed with the unpaired *t*-test: **P < 0.01, ****P < 0.0001.

We performed ELISA with several potyviruses to identify the specific binding of PVY and M13KO7. Potyvirus and potato viruses were confirmed by viral genome amplification and detection using reverse transcription-polymerase chain reaction (RT-PCR) and 1% agarose gel electrophoresis (Fig. 4A and C). The results (Fig. 4B) showed a correlation between the PVY and M13KO7. Eight potyviruses samples showed a negative reaction with M13KO7 bacteriophages as well as with healthy plant samples while reacting positively with PVY only. The other three potato virus samples, *Potato leafroll virus* (PLRV), *potato virus S* (PVS), and *potato virus X* (PVX), were found to be negative by bacteriophage ELISA (Fig. 4D), indicating that PVY detection with M13KO7 can be used for the diagnosis of PVY infection in field samples.

### Binding test of various bacteriophages to PVY

**Fig 5:**
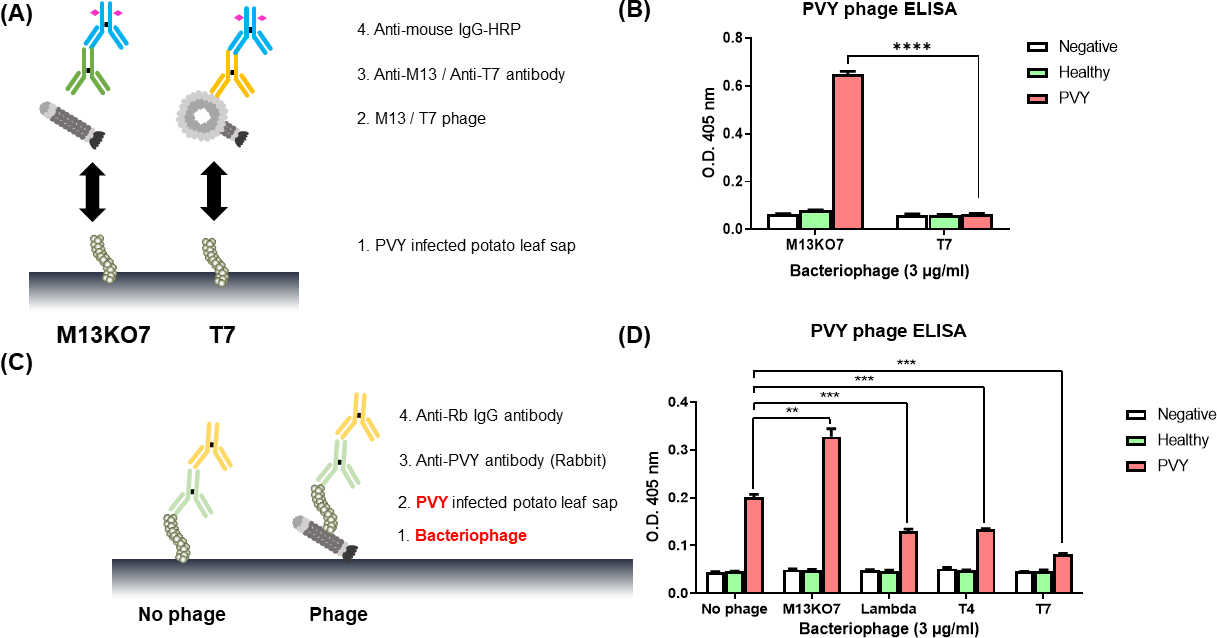
Several bacteriophages and Potato virus (PVY) ELISA. (**A**) Schematic of ELISA with M13KO7 and T7 bacteriophages. Monoclonal anti-M13-g8p antibody and anti-T7 antibody were used for detection. (**B**) Result of PVY detection bacteriophage ELISA measured at OD_405_ using a spectrophotometer. (**C**) Diagram of reverse ELISA of several bacteriophages. Carboxyl-coated microplates were used for capturing bacteriophages. (**D**) Result of reverse ELISA was measured at OD_405_ using a spectrophotometer. Except M13KO7, other bacteriophages inhibited PVY capture and detection (**P = 0.0019, ***P < 0.001, ****P < 0.0001 GraphPad Prism, GraphPad).

ELISA analyses were performed to determine whether other bacteriophages were capable of binding to PVY, and the corresponding schematic is shown in Fig. 5 (Fig. 5A and C). In the ELISA detection using T7 bacteriophages and the anti-T7 monoclonal antibody, PVY could not be detected in bacteriophages other than M13KO7 (Fig. 5B). When several other bacteriophages were coated on the microplate and PVY was added for reaction, M13KO7 captured more PVY particles than those captured without a bacteriophage or with any other bacteriophages (Fig. 5D).

### Binding test of PVY and other strains of M13 bacteriophage

**Fig 6:**
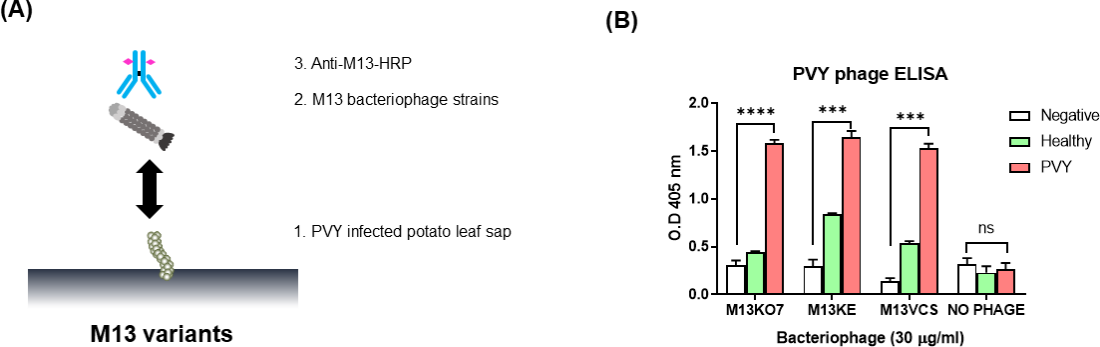
Several variants of M13 and Potato virus (PVY) ELISA. (**A**) Schematic of ELISA with M13 variants with PVY. (**B**) Result of PVY detection with M13 variants (M13KO7, M13KE, and M13VCS) and ELISA result measured at OD_405_. The statistical comparison was performed with the unpaired *t*-test: ***P < 0.001; ****P < 0.0001; ns, not significant.

ELISA analyses were performed to determine that M13 bacteriophage variants other than M13KO7 also have binding affinity to PVY (Fig. 6A). Each M13 bacteriophage variant was adjusted to the same concentration and used to treat negative, healthy potato leaf sap and PVY-infected potato leaf sap. As a result, other variants of M13 bacteriophage also showed binding affinity to PVY, of which three had a similar binding affinity to PVY (Fig. 6B). However, considering the binding affinity of other variants to negative and healthy control, M13KO7 was determined to be the best variant to detect PVY.

### Competitive ELISA

**Fig 7:**
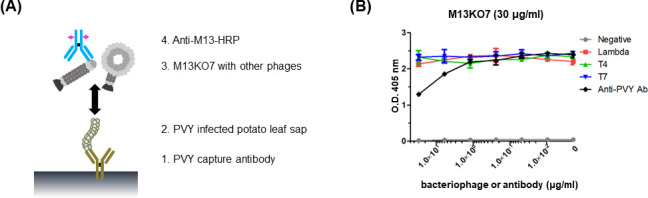
Competitive ELISA. (**A**) Competitive ELISA of M13KO7 and other bacteriophages. (**B**) Competitive ELISA results of M13KO7 and other bacteriophages (Lambda, T4, and T7) for Potato virus. Anti-PVY polyclonal antibody was used as a positive competitive control. M13 and other bacteriophages did not compete.

Competitive ELISA analyses were performed to investigate whether other bacteriophages can inhibit the interaction of M13KO7 and anti-M13 antibody. After coating the PVY samples on a microplate, M13KO7 and other bacteriophages were added to facilitate their simultaneous binding, and horseradish peroxidase (HRP)-conjugated anti-M13 antibody (Sino Biological Inc.) was used to detect the competition (Fig. 7A). As shown in Fig. 7B, other bacteriophages were not involved in the binding of PVY to M13KO7. Anti-PVY antibody was used as a positive control. Based on these observations, it was confirmed that bacteriophages having different structures from M13KO7 did not bind to PVY.

### Inhibition of M13KO7 with detergents and antibodies

**Fig 8:**
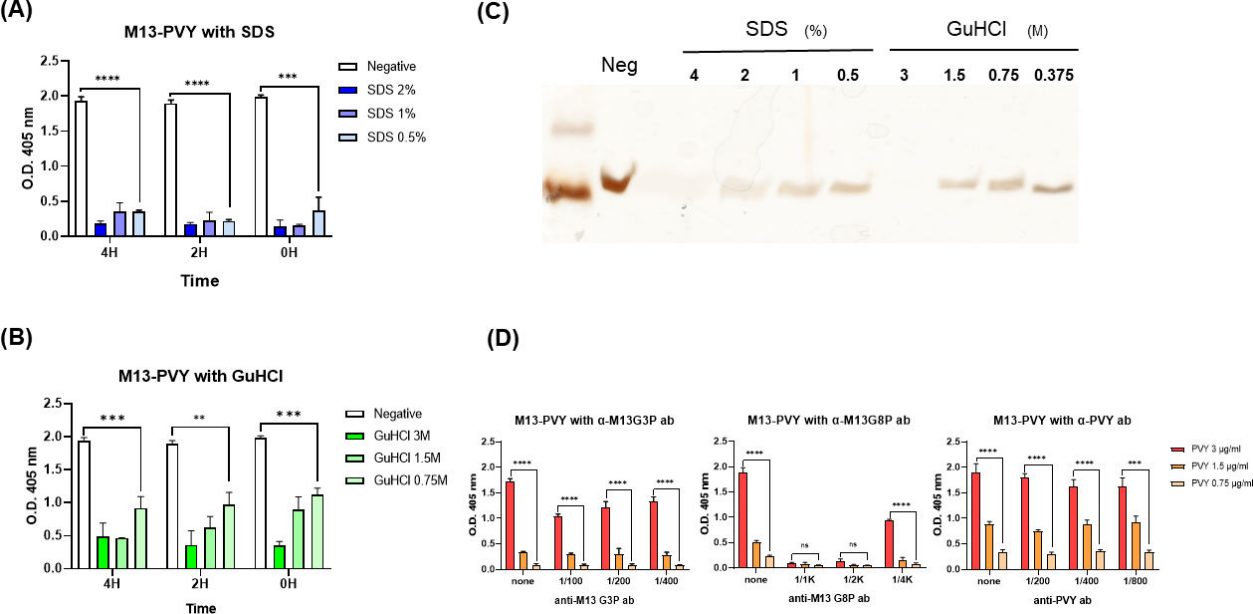
Inhibitory ELISA. (**A**) Result of ELISA for the virus (PVY) detection of M13KO7 treated with three SDS concentrations. (**B**) Results of ELISA for PVY detection of M13KO7 treated with three concentrations of GuHCl. (**C**) Silver-staining result of detergent-treated M13KO7. (**D**) Results of antibody-treated ELISA. M13KO7 was treated with anti-p3 antibody, anti-g3p antibody, and anti-PVY antibody. The statistic was analyzed using unpaired *t*-test: **P < 0.01; ***P < 0.001; ****P < 0.0001; ns, not significant.

ELISA analyses were performed to determine whether M13KO7 treatment with potential inhibitory antibodies or detergents affected its binding to PVY. After coating the PVY samples on a microplate, sodium dodecyl sulfate (SDS) (Fig. 8A) and guanidine hydrochloride (GuHCl) (Fig. 8B) were added to M13KO7 to destroy its structure. Silver staining was performed to confirm M13KO7 in the presence of detergents. The amount of g8p decreased after treatment with SDS and GuHCl (Fig. 8C), resulting in the decreased binding affinity of M13KO7 to PVY. In addition, M13KO7 was treated with an anti-M13 p3 antibody and anti-M13 g8p antibody. Furthermore, the anti-PVY antibody was treated using the same method (Fig. 8D). Our results indicated that treatment with anti-M13 p3 antibody and anti-PVY antibody did not considerably decrease the binding affinity of M13KO7, whereas treatment with anti-M13 g8p antibody markedly reduced the binding affinity. Therefore, the conformation of M13KO7 is important for binding with PVY, especially the gVIII protein (g8p).

### Robustness of M13KO7 at room temperature

ELISA analyses were performed to investigate robustness of M13KO7. M13KO7 was incubated at room temperature (RT, around 25°C) for several days to determine the stability of its binding to PVY at usual condition. RT-incubated M13KO7 showed no marked difference in binding activity to PVY (Fig. S2). This result indicated that M13KO7 is stable even when stored at RT for a long time, even M13KO7 is suggested to stock at −20°C.

### Comparative test of M13KO7and anti-PVY antibody to PVY

**Fig 9:**
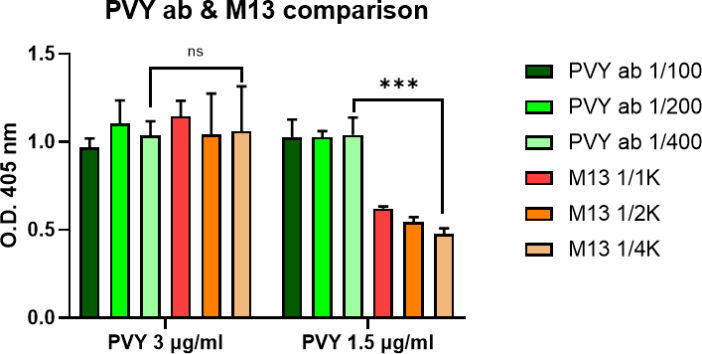
Comparative test of M13KO7 and anti-PVY antibody. Binding affinity of M13KO7 and PVY was compared to that of anti-PVY antibody and PVY. Half-diluted PVY virion was used as an antigen, and 10-fold serial diluted M13KO7 and anti-PVY antibody were tested by ELISA. The result of ELISA was measured at OD_405_. The unpair *t*-test was used to compare statistic: ***P < 0.001; ns, not significant.

ELISA was performed to test detection sensitivity of M13KO7 to PVY in comparison with anti-PVY antibody (Agdia, Inc., Elkhart, IN, USA). PVY capture antibody, PVY detection antibody, and M13KO7 were diluted in carbonate coating buffer and used for the test (Fig. 9). In these results, M13KO7 showed similar binding affinity to 3 µg/mL of PVY compared to that by PVY antibody although M13KO7 was diluted 10 times more than PVY antibody.

### Diagnostic sensitivity test of M13KO7 to PVY

**Fig 10:**
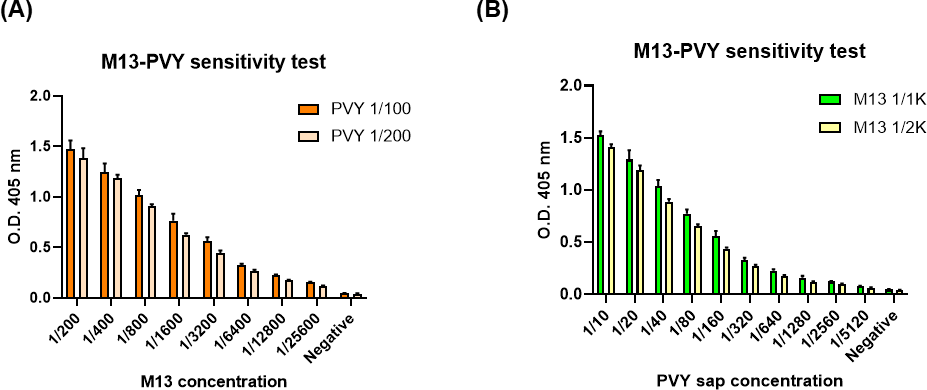
Sensitivity test of M13 KO7 and Potato virus (PVY) sap. (**A**) M13KO7 was twofold serial diluted and treated with two concentrations of PVY (1/100 and 1/200). (**B**) PVY sap was twofold serial diluted and treated with two concentrations of M13KO7 (1/1,000 and 1/2,000).

ELISA was performed to evaluate the sensitivity of M13KO7 to PVY by serial dilution binding test. First, PVY sap was extracted and diluted to 1/10 and 1/100 in general extraction buffer (GEB) and used for ELISA test. M13KO7 was 10-fold serial diluted in phosphate-buffered saline (PBS). The optical density (OD) at 405 nm value was found to be dependent on the concentration of M13KO7 (Fig. 10A). Otherwise, 1/10 of PVY sap was 10-fold serial diluted in GEB and used for antigen. Two concentrations of M13KO7 (1/1,000 and 1/2,000) were used for the ELISA test. OD_405_ value depended on the concentration of PVY sap though concentrations of M13KO7 did not show any significant difference (Fig. 10B). Limit of detection = 8.7 × 10^7^ and limit of quantitation = 2.6 × 10^8^ of PVY particles were determined by the ELISA tests, respectively.

## DISCUSSION

Current methods for diagnosing PVY include RT-PCR and ELISAs (28). However, RT-PCR requires RNA preparation and sophisticated equipment, such as a thermal cycler or UV transilluminator, making ELISA the preferred methodology. Although an indirect sandwich ELISA is commonly used, it is costly and vulnerable to physical and chemical changes. Furthermore, M13 bacteriophages can be easily propagated using *E. coli* and are resistant to non-aqueous media (29) and other physical or chemical changes (16) and non-polar organic solvents (17). Thus, detecting PVY using M13 can effectively replace diagnostic methods that use antibodies (Table S1).

We identified specific binding between PVY and M13KO7 using various plant viruses and bacteriophages. All potyviruses and potato viruses used for detection were confirmed using RT-PCR. M13KO7 bacteriophages showed only PVY binding among several plant viruses. However, to further confirm, reverse ELISA should be performed using antibodies specific to other potyviruses. In competitive ELISA, other bacteriophages such as Lambda, T4, and T7 bacteriophages did not affect the binding of PVY and M13KO7. However, we could not completely exclude the possibility of their binding to other epitopes of the antigens. Another filamentous bacteriophage, fd, is similar in structure to M13 and should be tested for its ability to bind to PVY. However, PVY and M13 bacteriophage have distinct interactions.

The PVY detection affinity of M13KO7 was confirmed under different pH, and it varied with change in pH. Bacteriophages pretreated with a strong acid (pH 0–2) showed high non-specific binding with healthy samples (data not shown). At a wide pH range (3–11), M13KO7 had the ability to specifically detect PVY, which is comparable to the pH range found to affect bacteriophage viability (Fig. 2A) (16).

In addition, M13KO7 was treated with detergents and antibodies to confirm their effect on the binding of M13KO7 and PVY. SDS and GuHCl were shown to inhibit the binding affinity of M13KO7 by disrupting the structure of the bacteriophage (30, 31). Treatment with anti-M13 p3 and anti-PVY CP antibodies had a negligible effect on the binding affinity of M13KO7, whereas anti-M13 g8p antibody reduced it considerably. These results suggest that the structure of M13KO7 is essential for the binding affinity and it may be related to g8p.

There are many advantages of using M13KO7 bacteriophages in diagnosing PVY. Bacteriophages have a much higher molecular weight than that of antibodies and are assumed to have higher stability with temperature and exposure to chemicals (Fig. 8; Fig. S1). In addition, the cost of producing bacteriophages for diagnosis of PVY is much lower than that for antibodies (32, 33, 34 – 37). As a result, PVY diagnosis can be performed at very low prices. Moreover, M13KO7 engineering could increase the convenience of detection. For example, a conjugation of fluorescence molecule like Fluorescein (FITC) can be used for diagnosis system development without secondary antibodies in further studies.

Many studies on filamentous bacteriophages, including M13, have identified structural similarities of plant viruses with helical capsids with bacteriophages (38 – 40). However, there is a lack of studies on interactions between plant viruses and bacteriophages. M13KO7 helper bacteriophages of mutated bacteriophages (19, 20, 22 – 24) are frequently used in bacteriophage displays for antibody screening. Binding between helper bacteriophages and target antigens interferes with antibody screening. Therefore, the interactions between helper bacteriophages and antigens need to be examined before antibody screening. When binding was tested between M13KO7 and PVY coat protein displayed on *Saccharomyces cerevisiae* (EBY100) by yeast surface display, no binding was observed (data not shown). The binding of PVY and M13KO7 is attributed to the interaction of structure or protein complexes, rather than the binding of each protein unit. Two unrelated viruses can interact with each other. In addition, other viruses not studied here may interact and bind. Unfortunately, we could not discover the specific molecular mechanism of M13KO7 and PVY binding.

### Conclusion

In this study, we investigated a new method for diagnosing the potato virus, PVY. PVY is one of the most important potato viruses because of its high infectivity and impact on crop yield. The method developed in this study involved the use of the M13KO7 bacteriophage to detect the PVY antigen or virus particles using ELISA. M13KO7 is easy to propagate by using *E. coli*, making this method more reasonable from an economic perspective. We also tested another potyvirus and bacteriophage as control samples of PVY and M13KO7, respectively. The control results showed no binding ability, and only PVY and M13KO7 were found to bind to each other. These results reveal two new virus-binding interactions and a novel system for the detection of PVY, making M13KO7 an efficient biological tool for the detection of PVY. However, the specific binding mechanism of PVY and M13KO7 is yet to be elucidated, which requires further research. These findings provide us with information that can be used for further studies on the interactions between viruses.

## MATERIALS AND METHODS

### PVY sample preparation

PVY-infected potato leaves were provided by the National Institute of Highland Agriculture. Potato leaf samples were stored in a deep freezer at −86°C. The potato leaf sap was homogenized in mesh bags with GEB [10 mM sodium sulfite, 2% (wt/vol) polyvinylpyrrolidone (MW 40,000), 0.2% (wt/vol) sodium azide, 2% (wt/vol) bovine serum albumin (BSA), and 2% (vol/vol) Tween-20 (Promega, Madison, WI, USA) with phosphate-buffered saline (PBS), pH 7.4] (41).

### M13KO7 bacteriophage propagation

M13KO7 bacteriophages (New England Biolabs, Ipswich, MA, USA) were propagated in *E. coli*. The titers of the propagated bacteriophages were measured as kanamycin-resistant colony-forming units (CFUs). XL1-blue (Agilent, Santa Clara, CA, USA) cells were grown at 37°C in 50 mL of 2TY containing 25 µg/mL tetracycline to an OD_600_ of 0.6. XL1-blue cells were inoculated with M13KO7 at a multiplicity of infection of 50. Cells were incubated for 30 min at 37°C without shaking for bacteriophage infection and then centrifuged for 10 min at 2,000 × *g*. The supernatant was discarded, and the pelleted cells were resuspended in 2TY broth containing 50 µg/mL kanamycin and 0.1% glucose. Bacteriophage propagation was allowed to proceed overnight at 30°C with shaking. The supernatant was then collected by centrifugation for 10 min at 2,000 × *g* and filtered using 0.22 µm pore Stericup filters (Merck Millipore, Billerica, MA, USA). M13KO7 bacteriophages were precipitated by adding 1/5 vol of PEG/NaCl (20% polyethylene glycol 6,000, 2.5 M NaCl) to the filtered supernatant, followed by mixing and incubation for 1 h on ice. Subsequently, the mixture was centrifuged at 2,000 × *g* for 1 h. The resulting supernatant was discarded, and the residual PEG/NaCl was removed by brief centrifugation. The bacteriophage pellet was resuspended in 1 mL of PBS and titrated with XL1-blue CFU on Lysogeny broth (LB) agar plates containing tetracycline and kanamycin.

### First ELISA method

To determine the binding activity of M13KO7, an ELISA was performed using a modified method (9). Briefly, ELISA plates were coated with PVY capture antibody (Agdia, Inc.), which was diluted to 0.1 µg/mL in carbonate coating buffer (15 mM sodium carbonate, 35 mM sodium bicarbonate, pH 9.6). The plates were incubated for 4 h at RT and rinsed three times with Tris-buffered saline (TBS) (pH 7.4) containing 0.1% (vol/vol) Tween-20 as a washing buffer (TBS-T). Homogenized PVY samples with GEB were incubated overnight at 4°C. The GEB and healthy leaf sap were used as controls. Following six rinses with TBS-T, the M13KO7 bacteriophages were serially diluted (fivefold dilution from 10 µg/mL) in blocking buffer [TBS-T with 3% (wt/vol) BSA] and added to the ELISA plates to bind PVY particles at RT for 2 h. After six additional washes, the plates were incubated with HRP-conjugated anti-M13 (1:1,000) (11793; Sino Biological Inc., Beijing, China) antibody at RT for 1 h. After six washes, 100 µL 3,3′,5,5′-tetramethylbenzidine (TMB) substrate was added for the enzymatic assay. The reaction was stopped by the addition of 1 M sulfuric acid for 2 min. OD was measured at 405 nm using a microplate spectrophotometer (Tecan Sunrise, Tecan, Switzerland).

### Second ELISA method

ELISA plate wells were coated for 4 h at RT with an anti-M13-g8p monoclonal antibody (1:1,000) (AS001; Antibody Design Lab, San Diego, CA, USA) in carbonate coating buffer. After three washes with TBS-T, bacteriophage particles were used to coat each ELISA plate at 3 µg/mL in blocking buffer at RT for 1 h. After rinsing three times, the homogenized PVY samples with GEB were incubated overnight at 4°C. The GEB and healthy leaf sap were used as controls. An additional plate was coated with potato samples without bacteriophage treatment. Following six washings with TBS-T, diluted PVY detection antibody (1 µg/mL) (Agdia, Inc.) with blocking buffer was added to the plates at RT for 1 h. After six additional washes, the plates were incubated with HRP-conjugated anti-mouse (1:1,000) (7076S; Cell Signaling Technology, Danvers, MA, USA) antibody at RT for 1 h. Again after six washes, 100 µL of TMB substrate (Agdia, Inc.) was added for the enzymatic assay. The reaction was stopped by the addition of 1 M sulfuric acid for 2 min. OD was measured at 405 nm using a microplate spectrophotometer.

### PVY detection by treatment of M13KO7 with heat and variable pH

Aliquots of sterilized distilled water were prepared with pH values ranging 3–14 using 1 N hydrochloric acid and 4 N sodium hydroxide. M13KO7 was added to the solutions of varying pH at a concentration of 300 µg/mL. Each tube was briefly vortexed, mixed, and incubated at RT for 1 h. Samples were neutralized with blocking buffer and verified with a pH meter (Orion 3-star Benchtop pH Meter; Thermo Fisher Scientific, Waltham, MA, USA) and then incubated at RT overnight. M13KO7 solution (3 µg/mL) was added to the potato sample-coated ELISA plates, as described above, and kept at RT for 2 h. The subsequent detection and development steps using HRP-conjugated anti-M13 antibody (Sino Biological Inc.) were performed using the first ELISA method described above.

To determine the effect of heat on PVY detection by denatured M13KO7, PVY samples were coated onto microplates as described above. M13KO7 was denatured in boiling water for 30 min, diluted in blocking buffer, and reacted with antigen-coated microplates. The results were then confirmed using HRP-conjugated anti-M13 antibody (Sino Biological Inc.).

### MALDI-TOF MS of M13KO7

The M13KO7 solution was titrated with sodium hydrate solution at pH 7 and pH 11–14 for 1 h at RT and diluted with distilled water to 3 mg/mL. Another M13KO7 solution (9 mg/mL) was boiled for 30 min. The bacteriophage samples (pH-treated, non-denatured, and denatured by heating) were transferred onto a MALDI plate using Millipore ZipTip μ-C18 tips (Merck Millipore) to remove the salts. The bacteriophage samples were analyzed using a Bruker Ultra-Flex I TOF/TOF mass spectrometer (Bruker, Billerica, MA, USA) with MS grade sinapic acid in 70% acetonitrile and 0.1% Trifluoroacetic acid as the matrix.

### Yeast two-hybrid experiment for identification of protein-protein relationships

A yeast two-hybrid (Y2H) experiment was designed to determine whether the protein subunits of M13KO7 and PVY were involved in binding. Five coat protein genes (gIII, gVII, gVII, gVIII, and gXI) of the M13KO7 bacteriophage and a coat protein gene of PVY were amplified using a primer set containing *attB*1 and *attB*2 sequences. The scFv encoding genes were amplified by PCR using primers (Table 1) in which the *AttB*1 and *AttB*2 sites were added to the 5′ and 3′ ends of the PCR product, respectively, for recombination site insertion for gateway cloning (42). To produce entry clones, the BP reaction was carried out using BP Clonase II Enzyme mix (Invitrogen, Carlsbad, CA, USA) and pDONR221 (Invitrogen) as donor plasmids. After the addition of proteinase K, the recombinant plasmids were transformed with DH5α and selected on LB media containing kanamycin (50 µg/mL). The subcloning of entry clones into destination plasmids, namely, pDEST22 (Invitrogen) was performed using LR Clonase II Enzyme mix (Invitrogen) for preparing prey and pDEST32 (Invitrogen) for bait expression clone. After the addition of proteinase K, the expression clones were transformed with DH5α and selected in selective medium (ampicillin for prey and gentamicin for bait). The expression clone sequences were analyzed using a DNA sequencing service (Macrogen, Seoul, Republic of Korea). The yeast (*Saccharomyces cerevisiae* strain MaV203) competent cell preparation was performed as described by Clontech (Tokyo, Japan) (43). Pre-culture was carried out by streaking cells onto yeast extract peptone dextrose (YPD) plates containing 2% dextrose (wt/vol) and then grown for 3 days at 30°C. A single colony was inoculated in fresh YPD and cultured to an OD_600_ of 1.5, diluted 10-fold in YPD media, and grown at 30°C to an OD_600_ of 0.4–0.6. The cells were harvested and washed with distilled water. The yeast was resuspended in sterilized TE/LiAc buffer (100 mM lithium acetate, 100 mM Tris-HCl, 10 mM EDTA, pH 7.5). The plasmid DNA and cells were mixed with carrier DNA (sheared salmon sperm DNA) and sterilized PEG/LiAc (40% PEG 4000, 100 mM lithium acetate, 100 mM Tris-HCl, 10 mM EDTA, pH 7.5) in a fresh tube. The tube was vortexed and incubated at 30°C for 30 min with shaking at 200 rpm. DMSO was added, and the tube was incubated for 15 min in a water bath at 42°C and left on ice for 1 min. Cells were then harvested at 15,000 × *g* for 5 s and resuspended in 1 × TE buffer and separated in synthetic defined (SD) media containing DO supplement (without leucine/tryptophan; SD-LT). After colonies were formed, plates were replicated onto selection plates (without leucine/tryptophan/histidine; SD-LTH) with 1 mM 3-amino-1,2,4-triazole.

**TABLE 1:** Nucleotide sequences of the primer sets used for the amplification of PVY CP and M13KO7 CPs for Y2H

Primer name	Primer sequence (5′−3′)
PVY-CP_attB1	GGG GAC AAG TTT GTA CAA AAA AGC AGG CTT CAT GGG ACA GGA CAC AAT C
PVY-CP_attB2	GGG GAC CAC TTT GTA CAA GAA AGC TGG GTC TTA CAT GTT CTT GAC TCC AAG
pIII_attB1	GGG GAC AAG TTT GTA CAA AAA AGC AGG CTT CAT GAA AAA ATT ATT ATT CGC AAT T
pIII_attB2	GGG GAC CAC TTT GTA CAA GAA AGC TGG GTC TTA AGA CTC CTT ATT ACG CAG
pVI_attB1	GGG GAC AAG TTT GTA CAA AAA AGC AGG CTT CAT GCC AGT TCT TTT GGG TAT
pVI_attB2	GGG GAC CAC TTT GTA CAA GAA AGC TGG GTC TTA TTT ATC CCA ATC CAA ATA AG
pVII_attB1	GGG GAC AAG TTT GTA CAA AAA AGC AGG CTT CAT GGA GCA GGT CGC GGA
pVII_attB2	GGG GAC CAC TTT GTA CAA GAA AGC TGG GTC TCA TCT TTG ACC CCC AGC
pVIII_attB1	GGG GAC AAG TTT GTA CAA AAA AGC AGG CTT CAT GAA AAA GTC TTT AGT CCT C
pVIII_attB2	GGG GAC CAC TTT GTA CAA GAA AGC TGG GTC TCA GCT TGC TTT CGA GGT G
pIX_attB1	GGG GAC AAG TTT GTA CAA AAA AGC AGG CTT CAT GAG TGT TTT AGT GTA TTC TT
pIX_attB2	GGG GAC CAC TTT GTA CAA GAA AGC TGG GTC TCA TGA GGA AGT TTC CAT TA

### Potyvirus RT-PCR detection

To identify the specific binding mechanism of PVY and M13KO7, we performed ELISA with several potyviruses. Potyvirus samples were obtained from PVGB (Republic of Korea), including *chili veinal mottle virus* (ChiVMV; PV-0897), *pepper mottle virus* (PepMoV; PV-1113), *pepper severe mosaic virus* (PepSMV; PV-1191), *potato virus A* (PVA; PV-0827), *potato virus V* (PVV; PV-0827), *tobacco vein mottling virus* (TVMV; PV-0251), and *watermelon mosaic virus* (WMV; PV-0393) in a lyophilized state. Additionally, *plum pox virus* (PPV) was obtained from infected leaves in Cheongdo-gun (Gyeongsangbuk-do, Republic of Korea) (44). Viral RNA was extracted using a Viral Gene-spin (iNtRON, Gyeonggi-do, Republic of Korea) according to the manufacturer’s instructions. To detect potyvirus genes, RT-PCR was performed using CellScript All-in-One 5 × First Strand cDNA Synthesis Master Mix (CellSafe, Yongin, Gyeonggi-do, Republic of Korea) following the manufacturer’s instructions and using the indicated primer sets (Table 2). The cDNA was amplified with AccuPower PCR master Mix (Bioneer, Daejeon, Republic of Korea) using a thermal cycler (T100; BioRad, California, USA) under the following conditions: 10 min at 96°C for pre-denaturation, followed by 35 cycles of thermal cycling (30 s at 96°C, 30 s at 55°C, and 1 min at 72°C), 10 min at 72°C for the final extension, and storage at 12°C. The amplified products were identified on 1.5% agarose gels containing ethidium bromide and visualized using a UV transilluminator.

**TABLE 2:** Nucleotide sequences of the primer sets used for RT-PCR detection for the potyviruses evaluated in this study^*a*^

Virus	Primer sequence (5′−3′)	Amplicon position	Reference
PVY	GGAAATGACACAATCGATG	8572–9372	This study
	CATGTTCTTGACTCCAAGTA		This study
PVA	TGTCGATTTAGGTACTGCTGGGAC	8636–8767	(45)
	TGCTTTGGTTTGTAAGATAGCAAGTG		(45)
PVV	AGAATCGTGTCCATCTTACAATGG	8269–8872	(46)
	TAAATTGACTCTGAGTTGCC		(46)
ChiVMV	GGAA(A/G)GC(G/A/T/C)CC(G/A/T/C)TA(C/T)AT	8421–9157	(47)
	CGCGCTAATGACATATCGGT		(47)
PepMoV	TGGGTCTGGCTCGATACGCATTTGA	9148–9640	(48)
	CTCGAGTTTTTTTTTTTTTTTTTT		(48)
PepSMV	GTGGAGCTTGACGATGAAC	8562–8714	(49)
	CTTATCAGTTGCTTTCACAT		(49)
PPV	ATGGCGAAGTCTCAGTTGCT	5454–5890	(44)
	AATTTGCGTGTTTTCGTTCC		(44)
TVMV	CATGTATGGAGTCAGTCCTG	5782–6208	(50)
	GCAAGCAGAAATTGGGT		(50)
WMV	AACGGTACATCTCCAGATG	9343–9641	(51)
	TTCATTTGTGCTATTGCTTCT		(51)

^*a*^ Amplicon position data were obtained from the following GenBank accession numbers: MF405303 for PVY, Z21670 for PVA, AJ243766.2 for PVV, AJ237843 for ChiVMV, NC_001517 for PepMoV, AM181350.1 for PepSMV, AB545926 for PPV, U38621.1 for TVMV, and AY437609 for WMV.

### Potyvirus ELISA detection

Potyvirus-infected plant samples were homogenized in GEB and coated onto ELISA plates at 4°C overnight. Following six TBS-T rinses, M13KO7 bacteriophages were diluted in blocking buffer and added to ELISA plates to bind PVY particles at RT for 2 h. After six additional washes with TBS-T, the plates were incubated with HRP-conjugated anti-M13 (1:1,000) antibody at RT for 1 h. After six more washes with TBS-T, 100 µL of TMB substrate was added for the enzymatic assay. The reaction was stopped by the addition of 1 M sulfuric acid for 2 min. The OD was measured at 405 nm using a microplate spectrophotometer.

### Potato virus ELISA detection

PLRV, PVS, and PVX potato leaves were kindly provided by the National Institute of Highland Agriculture. Viral RNA extraction and RT-PCR were performed as described above. The following three primer sets were used for the detection of potato virus: PLRV_F primer 5′-AGTACGGTCGTGGT-3′ and PLRV_R primer 5′-TTTGGGGTTTTGCAAAGC-3′ for PLRV, PVS_F primer 5′-GGAGCAGAGGCTCATCAGAT-3′ and PVS_R primer 5′-TGCCATTTGCTCAGTGTTCG-3′ for PVS, and PVX_F primer 5′-CAGTCCACCTG CTAACTGG-3′ and PVX_R primer 5′-TRACAGCTGCATCTAGGCT-3′ for PVX. The potato virus ELISA was performed using the same method as the potyvirus ELISA detection procedure.

### Bacteriophage ELISA

To confirm the binding of various bacteriophages and PVY, Lambda (BP-5203), T4 (BP-5204), and T7 (BP-5205) enterobacteria bacteriophages were collected from the Bacteriophage Bank of Korea (College of Natural Sciences, Hankuk University of Foreign Studies, Gyeonggi-do, Republic of Korea). The PVY virus samples were coated onto ELISA microplates, as described above. Diluted M13KO7 and T7 bacteriophages (1:1,000) were added to the plates and incubated at RT for 2 h. After three washes with TBS-T, the anti-T7 monoclonal antibody (1:1,000) (71530-3; Merck Millipore) and anti-M13-g8p monoclonal antibody (1:1,000) (Antibody Design Lab) were added to the respective plates and incubated at RT for 1 h. After six additional washes, the plates were incubated with HRP-conjugated anti-mouse (Cell Signaling Technology) antibodies at RT for 1 h. Again after six washes, TMB substrate (Agdia, Inc.) and 1 M sulfuric acid were used for the enzymatic assay. PVY capture testing was performed by coating the microplates with various bacteriophages. Next, 3 µg/mL M13KO7 and each of the three bacteriophages (Lambda, T4, and T7) were coated onto immobilizer microplates (Thermo Fisher Scientific) according to the manufacturer’s instructions. After three washes with TBS-T, the microplates were treated with blocking buffer at RT for 6 h. Then, the potato samples were homogenized and reacted with the prepared microplates. The detection procedure using a PVY antibody (Agdia, Inc.) was performed using the same process as the second ELISA method. Same experiment was performed with three M13 variants with concentration of 30 µg/mL (M13KO7, M13KE, and M13VCS).

### Competitive ELISA

Competitive ELISA experiments were performed using other bacteriophages to determine their interactions with PVY. The 96-well ELISA plates were coated with PVY capture antibody (Agdia, Inc.) at a concentration of 0.66 µg/mL in carbonate coating buffer. The plates were incubated for 4 h at RT and rinsed three times with TBS-T. The homogenized PVY samples with the GEB were incubated overnight at 4°C. After washing with TBS-T six times, M13KO7 (3 µg/mL) and fivefold serial dilutions of each of the three bacteriophages (Lambda, T4, and T7) with the blocking buffer were added to the plates. Anti-PVY polyclonal antibody (Agdia, Inc.) was used as a positive control. The microplates were incubated at RT for 2 h. After three washes, the plates were incubated with HRP-conjugated anti-M13 antibody (1:1,000) at RT for 1 h. After six washes, TMB substrate was added to the enzymatic assay. The reaction was stopped by the addition of 1 M sulfuric acid. The optical intensity was measured at 405 nm using a microplate spectrophotometer.

### Inhibition of M13KO7 with detergents and antibodies

Inhibitory ELISA experiments were performed using two anti-M13KO7 antibodies (anti-M13 pIII monoclonal antibody, E8033S; NEB, Ipswich, MA, USA) (anti-M13 bacteriophage coat protein g8p antibody, ab9225; Abcam, Cambridge, MA, USA), anti-PVY CP antibody (Agdia, Inc.), SDS, and GuHCl to determine the mechanism of interaction between M13KO7 and PVY. The 96-well ELISA plates were coated with purified and serially diluted PVY samples at a concentration of 1.25–5 µg/mL in carbonate coating buffer. The plates were incubated for 4 h at RT and rinsed three times with TBS-T. M13KO7 was mixed with each antibody (1:100–400 for g3p, 1:1,000–4,000 for g8p, 1:200–800 for PVY CP antibody), SDS (0.5%–2%), and GuHCl (0.75–3 M), in plates for 2 h at RT, and rinsed six times with TBS-T. After rinsing, the plates were again incubated with HRP-conjugated anti-M13 antibody (1:1,000) at RT for 2 h. After washing with TBS-T six times, the TMB substrate was added for the enzymatic assay. The reaction was stopped by the addition of 1 M sulfuric acid. The OD was measured at 405 nm using a microplate spectrophotometer.

### Silver staining for detection of M13KO7 g8p

Silver staining (Ezway Protein-Sliver staining kit; Komabiotech, Seoul, Republic of Korea) was performed to detect M13KO7 g8p after treatment with detergents. Then, 10% SDS-PAGE was used for verifying M13KO7. The gel was rinsed with deionized water (DIW) three times and treated with the first fixation buffer (50% DIW, 40% ethyl alcohol, and 10% acetic acid) overnight at RT. The gel was rinsed twice with the second fixation buffer (50% DIW and 50% ethyl alcohol) for 5 min each. Next, it was treated with the sensitizer buffer for 2 min and rinsed with DIW three times. Staining buffer was added for 20 min and rinsed with DIW twice followed by treatment with developer buffer for 5 min. The reaction was stopped by the addition of acetic acid for 10 min with agitation. The gel was rinsed with DIW for 30 min and observed with the naked eye.

### Robustness of M13KO7 at room temperature

ELISA was performed to investigate the robustness of M13KO7 after incubation at RT for 1, 3, and 7 days. The 96-well ELISA plates were coated with purified PVY samples at concentrations of 5 and 2.5 µg/mL in carbonate coating buffer. The plates were incubated for 4 h at RT and rinsed three times with TBS-T. Each RT-incubated M13KO7 was mixed with blocking buffer, treated into plates for 2 h at RT, and rinsed six times with TBS-T. After rinsing, the plates were incubated with HRP-conjugated anti-M13 antibody (1:1,000) and treated as mentioned above. The OD was measured at 405 nm using a microplate spectrophotometer.

## Data Availability

All data generated or analyzed during this study are included in the published article.

## SUPPLEMENTAL MATERIAL

Supplement data spectrum.01446-23-s0001.pdf Supplement data.

ASM does not own the copyrights to Supplemental Material that may be linked to, or accessed through, an article. The authors have granted ASM a non-exclusive, world-wide license to publish the Supplemental Material files. Please contact the corresponding author directly for reuse.
