# Supplementary material for: M13KO7 bacteriophage enables Potato Virus Y detection
Source: Microbiol Spectr. 2023 Oct 9;11(6):e01446-23. doi: 10.1128/spectrum.01446-23 (PMC10714723; doi:10.1128/spectrum.01446-23)
Supplement: Supplement data [file spectrum.01446-23-s0001.pdf]

Supplementary Table 1. Comparison of previous PVY detection methods and this research

	This study	R. Mishra et al., 2015	N. Petrov et al., 2015
Antigen	Purified PVY,<br>PVY infected potato leaf sap	PVY infected potato leaf<br>(No details provided)	Germs from potato<br>Seed tuber
ELISA method	Indirect ELISA (DAS, TAS)	Indirect ELISA (DAS)	Indirect ELISA (DAS)
Antibody	M13KO7 bacteriophage<br>30 µg/mL (or 1:1000)	Anti-PVY IgG (pAb)<br>1:1000	Anti-PVY IgG (pAb)<br>Not described
Measured OD	405 nm	405 nm	405 nm
Positive OD value	Purified PVY<br>~1.5–2.0<br>Potato leaf sap<br>~0.5–2.0	Potato leaf sap<br>~0.3–1.3	Potato germs<br>~1.8–3.0

Supplementary Fig. 1

(A)

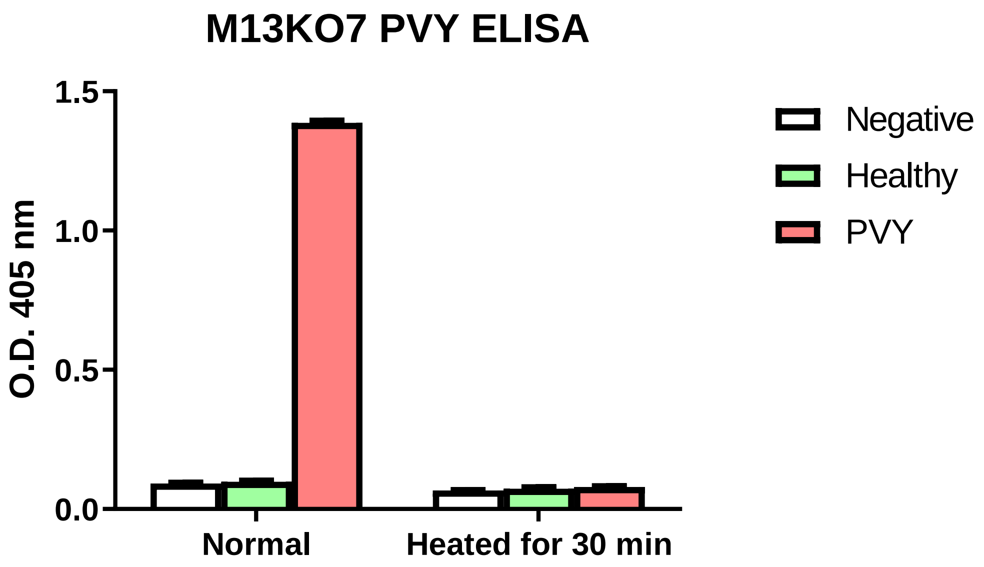

Supplementary Fig. 1

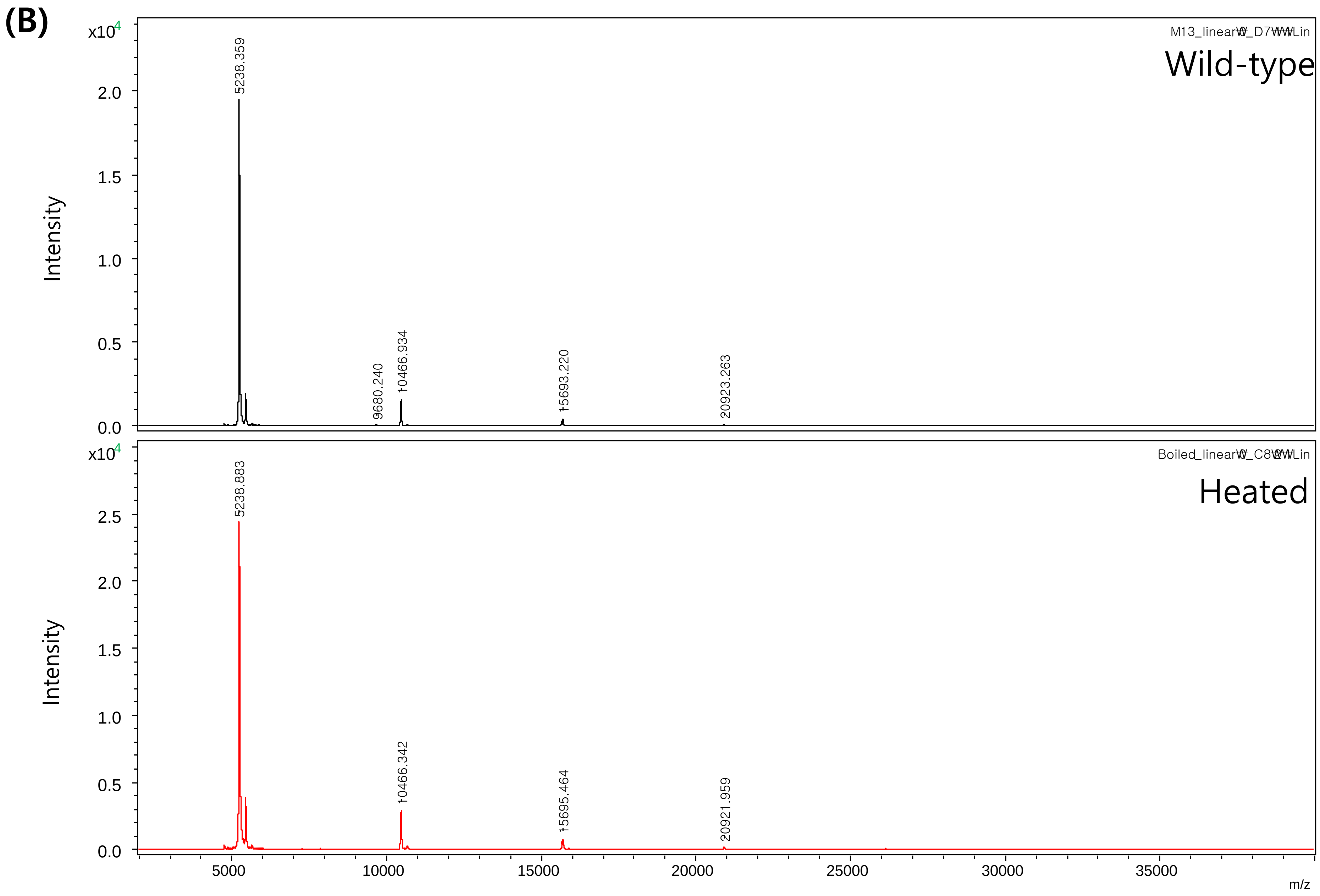

Supplementary Fig. 1. Effect of heat on M13KO7 ELISA for Potato virus Y (PVY). (A) PVY detection results of M13KO7 denatured with heating for 30 min and normal bacteriophage. Denatured bacteriophage has no PVY detection activity. (B) MALDI-TOF spectrum of thermally denatured M13KO7 and normal bacteriophage. Both peaks showed the same peak at 5239 m/z, and the peak patterns did not change, unlike the results of the pH changes.

Supplementary Fig. 2

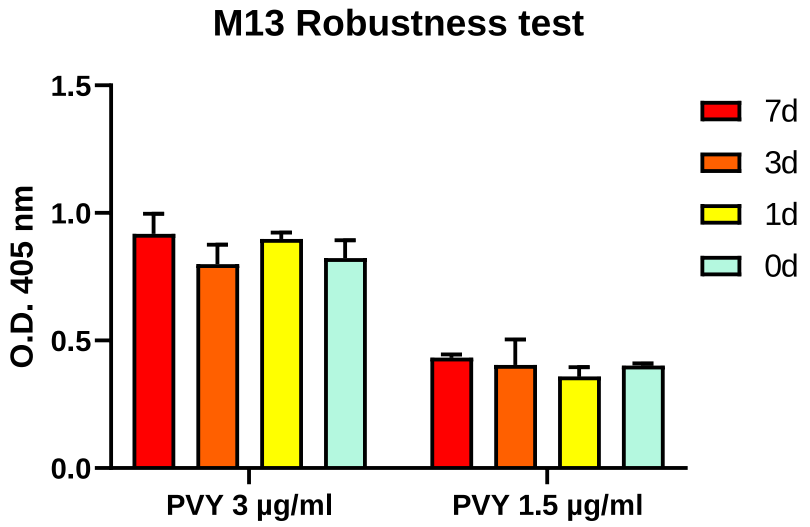

Supplementary Fig. 2. Robustness test of M13 bacteriophage. M13KO7 was incubated at room temperature for several days. After incubation, M13KO7 was used for detection of Potato virus (PVY), and the result of ELISA was measured at OD_{405} using a spectrophotometer.
